# Elongation factor-2 kinase regulates TG2/β1 integrin/Src/uPAR pathway and epithelial–mesenchymal transition mediating pancreatic cancer cells invasion

**DOI:** 10.1111/jcmm.12361

**Published:** 2014-09-12

**Authors:** Ahmed A Ashour, Nilgun Gurbuz, Sultan Neslihan Alpay, Abdel-Aziz H Abdel-Aziz, Ahmed M Mansour, Longfei Huo, Bulent Ozpolat

**Affiliations:** a Department of Experimental Therapeutics, The University of Texas, M.D. Anderson Cancer CenterHouston, TX, USA; b Department of Pharmacology and Toxicology, Faculty of Pharmacy, Al-Azhar UniversityCairo, Egypt; c Department of Molecular & Cellular Oncology, The University of Texas, M.D. Anderson Cancer CenterHouston, TX, USA; d Non-Coding RNA, The University of Texas, M.D. Anderson Cancer CenterHouston, TX, USA

**Keywords:** eEF-2K, Ca2+/calmodulin-dependent kinase III, tissue transglutaminase, rottlerin, Src, integrin, uPAR, MMP-2, EMT, pancreatic ductal adenocarcinoma

## Abstract

Pancreatic ductal adenocarcinoma is one of the lethal cancers with extensive local tumour invasion, metastasis, early systemic dissemination and poorest prognosis. Thus, understanding the mechanisms regulating invasion/metastasis and epithelial–mesenchymal transition (EMT), is the key for developing effective therapeutic strategies for pancreatic cancer (PaCa). Eukaryotic elongation factor-2 kinase (eEF-2K) is an atypical kinase that we found to be highly up-regulated in PaCa cells. However, its role in PaCa invasion/progression remains unknown. Here, we investigated the role of eEF-2K in cellular invasion, and we found that down-regulation of eEF-2K, by siRNA or rottlerin, displays impairment of PaCa cells invasion/migration, with significant decreases in the expression of tissue transglutaminase (TG2), the multifunctional enzyme implicated in regulation of cell attachment, motility and survival. These events were associated with reductions in β1 integrin/uPAR/MMP-2 expressions as well as decrease in Src activity. Furthermore, inhibition of eEF-2K/TG2 axis suppresses the EMT, as demonstrated by the modulation of the zinc finger transcription factors, ZEB1/Snail, and the tight junction proteins, claudins. Importantly, while eEF-2K silencing recapitulates the rottlerin-induced inhibition of invasion and correlated events, eEF-2K overexpression, by lentivirus-based expression system, suppresses such rottlerin effects and potentiates PaCa cells invasion/migration capability. Collectively, our results show, for the first time, that eEF-2K is involved in regulation of the invasive phenotype of PaCa cells through promoting a new signalling pathway, which is mediated by TG2/β1 integrin/Src/uPAR/MMP-2, and the induction of EMT biomarkers which enhance cancer cell motility and metastatic potential. Thus, eEF-2K could represent a novel potential therapeutic target in pancreatic cancer.

## Introduction

Pancreatic ductal adenocarcinoma (PDAC), the most common form of pancreatic cancer (PaCa), has a very poor prognosis with dismal survival rates of 2–3%, and ~6 months median survival with therapy 1,2. The major reason for poor prognosis is attributed to the extensive local tumour invasion, early systemic dissemination and metastasis, with the late development of clinical symptoms 3. The current therapies for PaCa offer very limited survival benefits, and surgical resection, for which only a minority (<20%) of patients qualify at time of diagnosis, is currently the only chance for cure, improving 5-year survival rates from <4%, if left untreated, to 25–30% after resection 4. Despite extensive clinical and scientific efforts and subtle progress over the years in terms of therapeutic strategies, the prognosis of this serious disease remains poor over the last years, and no major new treatment options have come forward from numerous clinical trials 1,5.

Pancreatic cancer, like all other cancers, is fundamentally a genetic disease caused by alterations in cancer-associated genes. In fact, much progress has been made in understanding the pathogenesis of genetic and molecular alterations and their role in pancreatic carcinogenesis 6. Progression from the non-invasive duct lesions to invasive cancer is associated with the accumulation of activating-mutations in the *K-RAS* oncogene (~90% of PDACs), inactivating-mutations in the tumour suppressors, *p53* and *SMAD4/DPC4*, as well as other genes mutations 1. The identifications of such alterations, along with new pro-oncogenic drivers are critical for better understanding the PaCa pathogenesis, and developing novel therapeutic strategies in order to prolong patient survival.

Metastasis is the major cause of patient death in almost all cancers 3,7,8, and it is a complex process regulated by multiple signalling pathways that are activated and/or overexpressed in tumours. The non-receptor protein tyrosine kinase-mediated signalling (*e.g*. Src), is one of these important pathways. The Src family is known to play critical roles in adhesion, migration and invasion as well as proliferation in cancer cells 9. To undergo metastasis into distant tissues, the cells have to penetrate and invade surrounding extracellular matrices; the process which is facilitated by the integrin family of cell adhesion molecules. Thus, altered integrin/Src activity can contribute to the neoplastic phenotype, and is proposed as an important target in cancer therapy 10. In addition, matrix metalloproteinases (MMPs), and urokinase plasminogen activator are important extracellular proteases derived from cancer cells that facilitate degradation of the extracellular matrix (ECM) and contribute to cancer invasion and progression 11. Urokinase-type plasminogen activator receptor (uPAR) is a cellular surface receptor that tightly binds to a serine protease urokinase-type plasminogen activator (uPA), a trypsin-like enzyme that plays a key role in regulating extracellular proteolysis, cell migration, adhesion and mobility 12. uPAR/uPA is endogenously expressed at high levels in advanced metastatic cancers, leading to plasminogen activation on the cell surface in a spatially restricted manner 13.

Epithelial-to-mesenchymal transition (EMT), is thought to be an important mechanism for promoting cancer invasion and progression of carcinomas 14. EMT is associated with lose of epithelial morphology and acquired mesenchymal characteristics. ZEB family of zinc finger transcription factors are essential master regulators of EMT 15. These transcription regulators trigger epithelial dedifferentiation by impairing the expression/function of E-cadherin 16,17. The epithelial adhesion protein, E-cadherin, is an active suppressor of invasion and growth of many epithelial cancers, and its down-regulation is considered a hallmark of EMT 18,19.

Recently, most of the efforts were focused on designing new targeted therapeutic approaches for inhibiting the conventional therapies-resistant and the highly invasive phenotype of PaCa. With this motivation, we recently found that the eukaryotic elongation factor-2 kinase (eEF-2K), also known as Ca2+/calmodulin-dependent kinase III, is highly overexpressed in PaCa cell lines 20. eEF-2K is a negative regulator of peptide elongation *via* phosphorylation of eEF2, a key component of the translation machinery 21,22. eEF-2K is activated during mitosis, hypoxia, metabolic stress, nutrients-deprivation as well as by mitogens and growth factors 23–25. In addition, eEF-2K regulates autophagy, which is an important mechanism that allows the cell to conserve energy or direct energy to other cellular functions. Thus, eEF-2K could act as a pro-survival kinase that promotes the signalling pathways related to cell growth, survival and drug resistance 26. However, the mechanisms by which eEF-2K mediates such signalling remain to be elucidated.

Here, we aimed to explore the role of eEF-2K in PaCa cells invasion and migration, and to investigate the involved downstream signalling pathways. We investigated the potential of targeting eEF-2K by rottlerin, a Kmala Tree-derived compound with anti-cancer activity. We previously showed that rottlerin, down-regulates eEF-2K protein and mRNA expression, independently of PKC-δ, in PaCa cells 20, providing a useful tool to investigate the effects of eEF-2K down-regulation. In this study, we provided a novel insight into the involvement of eEF-2K in the invasive/metastatic phenotype and related signalling in PaCa. Importantly, our data indicated that eEF-2K, regulates the expression of tissue tranglutaminase (TG2), the multifunctional protein which is abundantly overexpressed in highly metastatic cells 27, as well as β1 integrin/Src/uPAR/MMP-2 signalling. Moreover, eEF-2K regulates EMT, through modulation of the TCF8/ZEB1, Snail and claudins, further linking eEF-2K to malignant tumour progression. Collectively, our data suggest that eEF-2K is a novel mediator of PaCa cells invasion signalling and EMT drivers that are associated with a poor prognosis in PaCa.

## Materials and methods

### Cell lines, culture conditions and reagents

The human PaCa cell lines employed were obtained from American Type Culture Collection (Manassas, VA, USA). PANC-1 and MIAPaCa-2 cells were cultured in DMEM/F12 supplemented with 10% FBS. All media contain penicillin and streptomycin (100 units/ml). Cells were maintained at 37°C in a humidified atmosphere containing 5% CO_2_/95% air, and were used between passages 4 and 15. Rottlerin was purchased from (Sigma-Aldrich, St. Louis, MO, USA), dissolved in DMSO and directly added to the cell cultures at indicated concentrations (μM). Control cells were treated with DMSO alone.

### Transfections with siRNA

siRNA targeting eEF-2K (Sigma-Aldrich) was designed using siRNA-designing software (Qiagen, Valencia, CA, USA): eEF-2K siRNA#1, 5′-GCCAACCAGUACUACCAAA-3′ 20,26. A previously published eEF-2K siRNA: eEF-2K siRNA#2, 5′-AAGCUCGAACCAGAAUGUC-3′ 28, control non-silencing siRNA (5′-AAUUCUCCGAACGUGUCACGU-3′) 20,29, siRNA targeting Src (Sigma-Aldrich), and siRNA targeting TG2 (Qiagen) were also employed. Cells were transfected with either siRNA, at a final concentration of 50 nM for 72 hrs, using HiPerFect Transfection Reagent (Qiagen) according to the manufacturer’s protocol. The concentrations of siRNAs were chosen based on dose–response studies.

### Packaging of pCDH constructs into the viral particles and the production of lentivirus

eEF-2K gene 26 was subcloned into pCDH lentiviral vector (System Biosciences SBI, Frederick, MD, USA) from pcDNA3.1-eEF-2K vector. The pCDH-eEF-2K lentiviral vector and its packaging plasmid mix (psPAX2 and pMD2.G) were transfected into HEK293T packaging cells (SBI) by lipofectamine transfection (Invitrogen/Life Technologies, Carlsbad, CA, USA). Briefly, mammalian 293T cells (in rapid replication state) were seeded (1 × 10^6^) into 25 cm^2^ flask 1 day before transfection, in growth medium without antibiotics to reach around 90% confluence on the next day. In 0.5 ml Opti-MEM, the pCDH-eEF-2K vector (10 μg), was mixed with the packaging plasmid psPAX2 (10 μg) and the envelope plasmid pMD2.G-VSV-G (1 μg) to get a ratio of 1:1:0.1. These plasmid DNAs were combined with a diluted Lipofectamine 2000 (50 μl in 0.5 ml Opti-MEM), and incubated at room temperature for 20 min. Then, the cells were cotransfected with the Lipofectamine-DNA mixture. Within 18 hrs of incubation, the transfection medium was replaced with fresh culture medium. 48 hrs later, the lentivirus-containing medium was collected and centrifuged at 500 × g for 5 min. to pellet the cell debris, and the supernatant was passed through a 0.45-μm filter. The lentiviral particles is stored at −80°C, and thawed just before transduction of target cells, to avoid lentivirus loss as a result of multi-cycle of freeze/thaw. The target cells were infected with fresh lentivirus-containing medium (supplemented with 8 μg/ml polybrene) for 48 hrs, and subjected to puromycin selection. Similarly, control lentivirus (empty vector) were produced and transduced into target cells.

### Transduction of PANC-1 cells with eEF-2K lentiviral expression vectors

To establish stable integration of the viral expression construct into PANC-1 cells genomic DNA, to get clones stably expressing human eEF-2K gene, PANC-1 cells were transuded with pCDH-eEF-2K lentiviral plasmid, and the stable clones were selected against puromycin. Briefly, PANC-1 cells were seeded into 6-well plate (2 × 10^5^ cells/well) and incubated overnight. Next day, the medium was replaced by a serum-free medium, supplemented by polybrene (EMD Millipore Corporation, Billerica, MA, USA) at final concentration of 8 μg/ml. Then, 100 μl of the concentrated packaged pseudo-viral particles (pCDH-eEF-2K and empty control vectors), that prepared as described above, were added to the corresponding wells. The plate was centrifuged at 500 × g for 2 hrs, to enhance virus infection, and incubated at 37°C, 5% CO_2_ for about 5 hrs. After cell transduction, 1 ml medium containing FBS and antibiotics were added to the culture and the cells were incubated for 48 hrs. Then, the old media were replaced by fresh media containing puromycin (3 μg/ml; Invitrogen/Life Technologies), and the cells were re-incubated for an additional 48–72 hrs. Multiple stable clones were used to rule out potential clonal effects, and eEF-2K gene expression was determined and verified by Western blotting.

### Matrigel invasion assay

The cells were treated with indicated rottlerin concentrations or the vehicle (DMSO) or transfected with 50 nM of indicated siRNAs, and 24 hrs later, live cells were collected, counted and fixed numbers of the viable cells (1.5 × 10^4^ cells), were seeded onto Matrigel-coated Transwell filters (8-mm pore size) in Matrigel invasion chambers (BD Biosciences, San Jose, CA, USA). Similarly, cells transduced with eEF-2K lentiviral expression vectors or control vector were incubated with or without indicated rottlerin concentrations, and Matrigel invasion assay was employed as above. The number of cells that invaded the lower side of the membrane after 48 hrs was determined by counting cells in a minimum of four randomly selected areas. The experiment was performed in triplicate and the results were reported as a mean number ± SD.

### Migration assay

*In vitro* wound-healing assay was used to assess cell motility and the ability to migrate. PANC-1 cells were plated in 6-well plates (5 × 10^5^ cells/well), and cultured in medium containing 10% FBS to achieve a nearly confluent cell monolayer. A scratch was then carefully made on the cell layer using a 10 μl sterile micropipette tip, and any cellular debris was removed by washing with PBS to remove floating cells. The wounded monolayers were then incubated with the indicated rottlerin concentrations or the vehicle (DMSO) or transfected with 50 nM of indicated siRNAs. Similarly, eEF-2K-transduced cells and control vector-transduced cells were incubated with or without indicated rottlerin concentrations. Immediately after the treatments, the cells were photographed using a phase-contrast microscope (Nikon Instruments Inc., Melville, NY, USA), to determine the wound width at time 0. The cultures were continued, and the cells were photographed again after 12 hrs and after 24 hrs of wounding the cell layer. The wound healing was visualized by comparing photographs taken at 0 hr with those taken at 12 and 24 hrs later, and analysed for the distance migrated by the leading edge of the wound at each time-point. The distance travelled by the cells was determined by measuring the wound width at time 12 and 24 hrs, and subtracting it from the wound width at time 0. The values obtained were then expressed as % migration, setting the gap width at 0 hr as 100%. Three experiments were done in triplicate.

### Western blot analysis

Cells were seeded in 25-cm^2^ culture flasks (0.5 × 10^6^ cells/flask). After treatments, the cells were collected, centrifuged, washed twice in ice cold PBS and whole-cell lysates were obtained by suspending the cells in a lysis buffer at 4°C. Lysates were centrifuged at 13,000 × g for 10 min. at 4°C, and the supernatant fractions were collected. Total protein concentration for each sample was determined by a detergent compatible protein assay kit (Bio-Rad, Hercules, CA, USA), and Western blotting was performed as described before 20. The membranes were probed with the following primary antibodies (diluted in TBS-Tween 20 containing 2.5% dry milk, and incubated at 4°C overnight); eEF-2K, p-eEF2 (Thr-56), eEF2, p-Src (Tyr-416), Src, β1 integrin, uPAR, MMP-2, Claudin-1, Snail, TCF8/ZEB1, E-cadherin, Zona occludins (ZO)-1, N-cadherin, Slug, Vimentin, β-Catenin, α-β-Tubulin (Cell Signaling Technology, Danvers, MA, USA); c-Myc, Fibronectin (Santa Cruz Biotechnology, Santa Cruz, CA, USA); Collagen type I (Southern Biotechnology, Birmingham, AL, USA) α-SMA, TG2 (Abcam; Cambridge, MA, USA) and β-actin (Sigma Chemical, St. Louis, MO, USA). After being washed with TBS-T, the membranes were incubated with horseradish peroxidase-conjugated anti-rabbit, antimouse secondary antibody (Cell Signaling Technology) or rabbit anti-goat antibody (Invitrogen/Life Technologies). Chemiluminescent detection was performed with Chemi-glow detection reagents (ChemiGlow West Chemiluminescence Substrate Kit, ProteinSimple, Santa Clara, CA, USA). The blots were visualized with a FluorChem 8900 imager and quantified by a densitometer using the image analysis program (ImageJ 1.48s processing software, National Institutes of Health, Bethesda, MD, USA). All experiments were independently repeated at least twice.

### RNA isolation and reverse transcriptase-polymerase chain reaction (RT-PCR) analysis

Total cellular RNA was isolated from the collected cells with TRIzol® Reagent (Invitrogen/Life Technologies), and cDNA was obtained from 1 μg of total RNA using RevertAid First Strand cDNA Synthesis Kit (Thermo Scientific, Waltham, MA, USA). The cDNA for TG2, β1 integrin and GAPDH were amplified by PCR, using Platinum® Taq DNA Polymerase kit (Invitrogen/Life Technologies), with specific primers. Briefly, 2 μl of the total 20 μl of reverse-transcribed product were used for PCR in 1X PCR buffer containing 1.5 mM MgCl_2_, 200 μM deoxynucleotide triphosphates (dNTPs), 1 unit of Platinum Taq polymerase, and 0.2 μM of each TG2 primers, β1 integrin primers (Integrated DNA Technologies, IDT, Coralville, IA, USA), or GAPDH-specific primers (Thermo Scientific, Waltham, MA, USA). The sequences of the sense and anti-sense TG2 primers are 5′-TAAGAGATGCTGTGGAGGAG-3′; and 5′-CGAGCCCTGGTAGATAAA-3′, respectively. The sequences of the sense and anti-sense β1 integrin primers are 5′-CCTACTTCTGCACGATGTGATG-3′; and 5′-CCTTTGCTACGGTTGGTTACATT-3′, respectively. The cDNA samples were incubated at 94°C (2 min.) to denature the template and activate the enzyme. This step was followed by 35 cycles of PCR amplification (in each cycle, the samples were incubated at 94°C for 30 sec., 60°C for 30 sec. and 72°C for 60 sec.) with an additional cycle at 72°C for 5 min. The amplified reaction products were analysed on a 1.2% agarose gel containing ethidium bromide. The cDNA synthesis was verified by detection of the GAPDH transcript, which was used as an internal control.

### Reverse phase protein arrays (RPPA)

The control vector- and eEF-2K vector-transfected PANC-1 cells (0.5 × 10^6^ cells/2 ml media) were seeded in 6-well plate. After 48 hrs incubation, the cells washed twice with PBS, and 150 μl of the lysis buffer [1% Triton X-100, 50 mM Hepes (pH 7.4), 150 mM NaCl, 1.5 mM MgCl_2_, 1 mM EGTA, 100 mM NaF, 10 mM Sod.pyrophosphate, 1 mM Na_3_VO_4_ and 10% glycerol, containing proteinase and phosphatase inhibitors (Roche Applied Science, Indianapolis, IN, USA)] were added to each well. The cell lysates were collected, and the protein concentration was determined by routine Bradford assay (Bio-Rad), and adjusted to 1.5 mg/ml in each sample. Cell lysates were mixed with 4× SDS/2-ME sample buffer (40% glycerol, 8% SDS, 0.25 M Tris-HCl, pH 6.8; with 10% 2-mercaptoethanol added before use) in 3:1 ratio. For RPPA processing, protein cell lysates were twofold-serial diluted for five dilutions (1:1, 1:2, 1:4, 1:8 and 1:16 dilutions) and arrayed on nitrocellulose-coated glass slide (FAST Slides, Whatman Inc, Sanford, ME, USA) in 11 × 11 format, and samples were probed with antibodies (mouse, rabbit or goat). Slides were scanned, and the density of each identified spot was quantified by MicroVigene. Each spot on the array slide represents a certain dilution of the lysate of a particular sample. Relative protein levels for each sample were determined by interpolation of each dilution curves, from the ‘standard curve’ (supercurve, constructed by Bioinformatics) of the slide (antibody). All the data points were normalized for protein loading and transformed to linear value. These values are transformed to Supercurve Log2 value, and then median-centred for Hierarchical Cluster analysis 30. Technical RPPA duplicate was applied to all samples at the RPPA Process Core Facility at MD Anderson Cancer Centre, and average values of proteins levels identified from a pool of 225 primary antibodies, were provided.

### Statistical analysis

The data were expressed as the means ± SD of three independent experiments, and the statistical analysis was performed with the Student’s *t*-test, to determine statistical significance. *P* values less than 0.05 were considered statistically significant and are indicated by an asterisk.

## Results

### Targeting eEF-2K impairs invasion of PaCa cells

**Figure 1:**
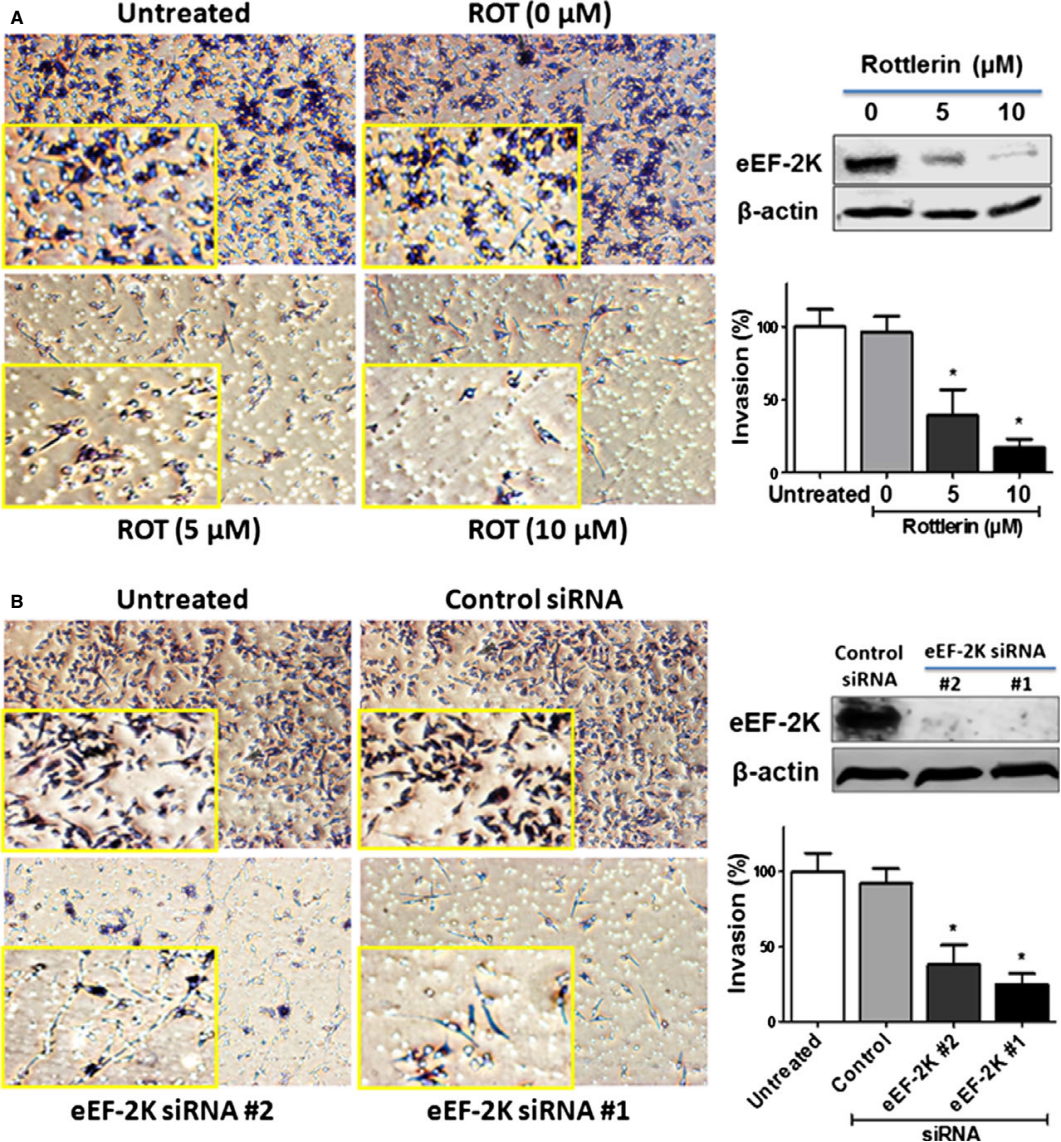
Effect of rottlerin treatment and siRNA-mediated eukaryotic elongation factor-2 kinase (eEF-2K) knock-down on invasion of PANC-1 cells. Cells were treated with indicated rottlerin concentrations or the vehicle (DMSO; **A**) or transfected with indicated siRNAs (**B**), and seeded onto Matrigel-coated Transwell filters in Matrigel invasion chambers. The number of the invaded cells was determined in a minimum of four randomly selected areas. Magnification, 100X. The Western blots show the down-regulation of eEF-2K after rottlerin treatment or after cells transfection with two different sequences of eEF-2K siRNAs. The histograms show the percentages of the invaded cells. Data are expressed as mean of the percentages of invasion ±SD of three experiments. *P < 0.05 *versus* control cells.

A major hallmark of PDAC is extensive local tumour invasion and early systemic dissemination 31. First, we investigated the effect of rottlerin, as an eEF-2K inhibitor, on the invasion of PANC-1 cells, by performing *in vitro* invasion assay using Matrigel-coated Boyden chambers. This assay mimics the *in vivo* invasion process and measures the number of cancer cells migrating through a basement membrane matrix towards media containing chemo-attractants 32. We have previously showed that rottlerin treatment down-regulates eEF-2K at a concentration range of 5–10 μM 20. Here, we show that rottlerin treatment, at 5 and 10 μM, markedly reduced invasion of PANC-1 cells by about 61% and 83%, respectively (Fig. 1A). To show direct link between eEF-2K and invasion, we further induced knock-down of eEF-2K by two different siRNA sequences 20,26, and performed the *in vitro* invasion assay. As expected, specific knock-down of eEF-2K by siRNAs, #1 and #2, reduced the invasion of PANC-1 cells by about 75% and 62%, respectively (Fig. 1B). These results suggest that eEF-2K is involved in regulation of PaCa invasion, and its down-regulation markedly impairs the invasion capacity of PaCa cells.

### eEF-2K expression induces PaCa cells invasion and suppresses rottlerin’s anti-invasive effect

**Figure 2:**
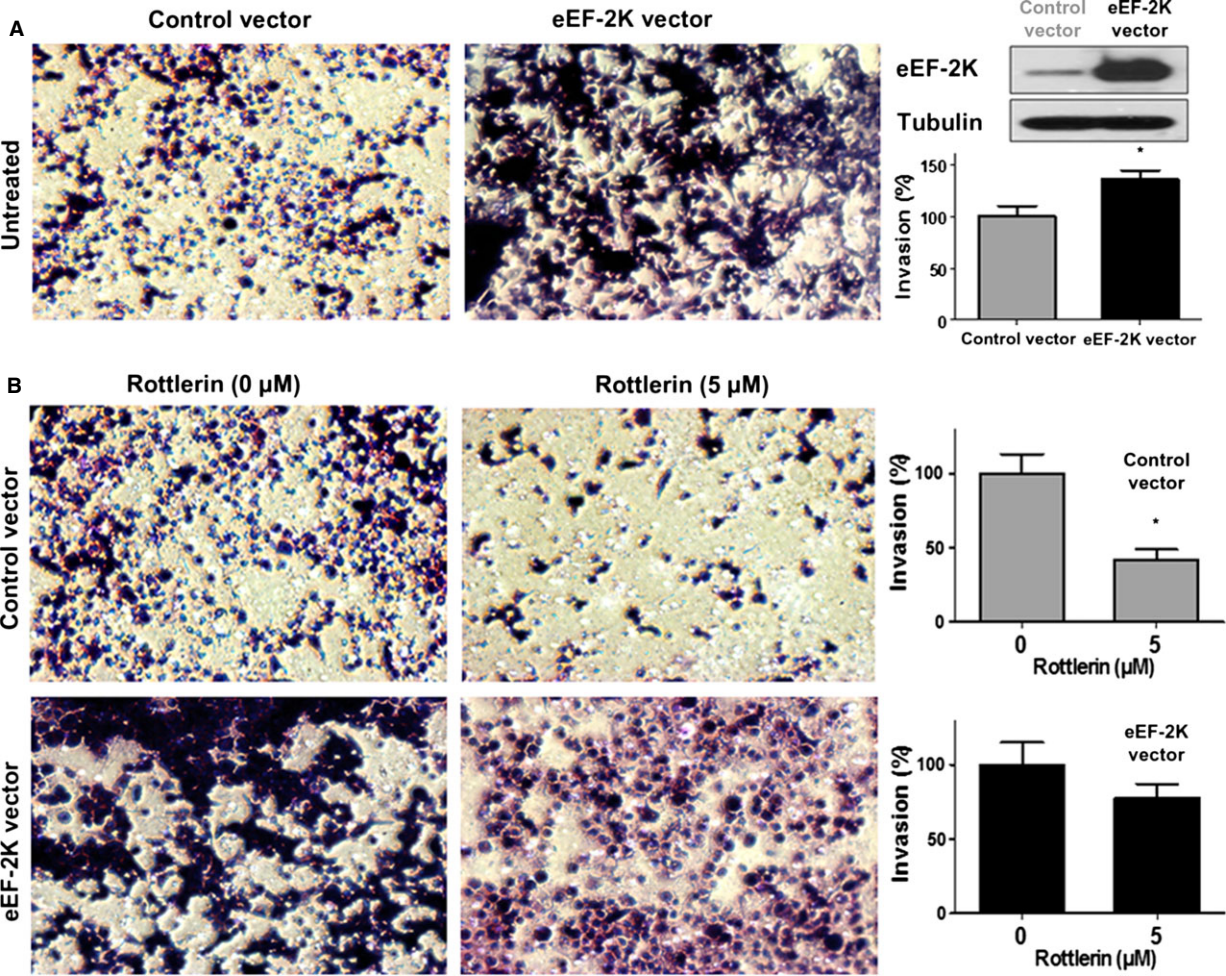
Eukaryotic elongation factor-2 kinase (eEF-2K) overexpression augments PANC-1 cells invasion, and suppresses rottlerin-mediated inhibition of cellular invasion. The cells were stably transfected with pCDH-eEF-2K lentiviral vector or empty control vector. The untreated cells (**A**) or the cells treated with indicated rottlerin concentrations (**B**) are seeded onto Matrigel-coated Transwell filters in Matrigel invasion chambers, and the number of the invaded cells was determined in a minimum of four randomly selected areas. Magnification, 100X. The Western blots show the relative expression of eEF-2K after transducing the cells with the indicated vectors. The histograms show the percentages of the invaded cells. Data are expressed as mean of the percentages of invasion ±SD of three experiments. *P < 0.05 *versus* control cells.

Given the suggested role of eEF-2K in PaCa cell invasion, we further investigated whether overexpression of eEF-2K gene promotes cell invasion and protects the cells from rottlerin-mediated inhibition of invasion. Thus, we overexpressed human eEF-2K gene in PANC-1 cells, using pCDH-eEF-2K lentiviral expression construct, and established stable clones of PANC-1 overexpressing eEF-2K, as described in Materials and Methods. The *in vitro* invasion assay was employed using control empty vector- and eEF-2K expression vector-transduced PANC-1 cells. As shown in Figure 2A, eEF-2K overexpression led to a significant increase (~36%) in the number of the invading cells that migrated through the matrigel, compared to the control vector-transduced cells. These results further implied that eEF-2K expression plays a role in the invasion of PaCa cells. To reveal if rottlerin-induced eEF-2K down-regulation mediates the rottlerin’s effect on PaCa invasion, we examined the invasion of both control vector- and eEF-2K vector-transduced cells, after rottlerin or DMSO treatment. The overexpression of eEF-2K led to a marked suppression of the rottlerin-induced inhibition of cell invasion capacity, compared to the control vector-transduced cells (Fig. 2B), suggesting that eEF-2K is a major target of rottlerin, whose down-regulation is a critical to mediate rottlerin-induced inhibition of PaCa cells invasion. Taken together, this data suggest that eEF-2K-regulated signalling could mediate the acquisition of invasive and metastatic potential of PaCa cells.

### eEF-2K promotes PaCa cells motility/migration and suppresses rottlerin-induced inhibition of the cells migratory capacity

**Figure 3:**
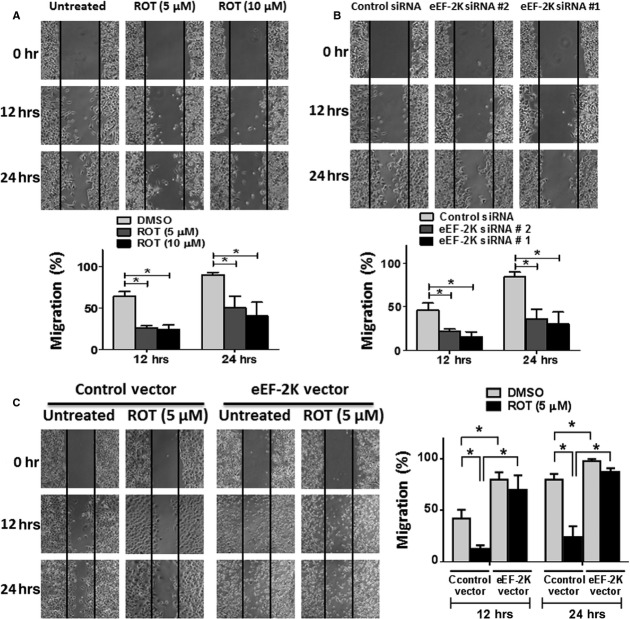
Involvement of eukaryotic elongation factor-2 kinase (eEF-2K) in pancreatic cancer (PaCa) cell motility. (**A** and **B**) The effects of rottlerin treatment and siRNA-mediated eEF-2K knock-down on PANC-1 cells migration capability were analysed by the wound-healing assay. A single scratch was made in the centre of the confluent cell monolayer, and the wounded monolayers were incubated with the indicated rottlerin concentrations or the vehicle (DMSO; **A**) or transfected with indicated siRNAs (**B**). The wounds repair was monitored for 24 hrs and visualized microscopically with original magnification ×100. Images were taken immediately (0 hr) and after 12 and 24 hrs of scratching the cultures. (**C**) eEF-2K overexpression augments PANC-1 cells migration, and suppresses rottlerin-mediated inhibition of cellular migration. The stably transfected cells with pCDH-eEF-2K lentiviral vector or empty control vector were treated as in (**A**), and the width of the wound areas were monitored as above. The histograms show the percentages of the cells migration, and the data are expressed as mean of the percentages of migration ±SD of three independent experiments. * represents significant difference between indicated groups (*P* < 0.05).

Because we show the regulatory role of eEF-2K of the invasion capacity of PaCa cells (at 48 hrs), we next examined the involvement of eEF-2K in mediating cell motility using the scratch assay at earlier time-points (12 and 24 hrs). The analysis revealed that the distance covered by migrating cells (at 12 and 24 hrs) was significantly decreased when PANC-1 cells were exposed to rottlerin or depleted from eEF-2K, using 2 different siRNAs, compared to cells exposed to the vehicle (DMSO), or non-silencing control siRNA, respectively (Fig. 3A and B). Conversely, the eEF-2K-transduced cells exhibited an enhanced migratory capacity to the wounded areas in both DMSO-treated and rottlerin-treated groups compared with the empty vector-transduced cells (Fig. 3C). Clearly, overexpression of eEF-2K suppressed rottlerin-induced inhibition of cell migration, comparing with the control cells. Overall, theses result indicates a correlation between PANC-1 motile behaviour and eEF-2K expression. It also, suggests that the rottlerin-induced inhibition of migration is mediated, at least partially, *via* eEF-2K down-regulation.

### eEF-2K-mediated regulation of TG2 could account for promoting PaCa cells migration/invasion

**Figure 4:**
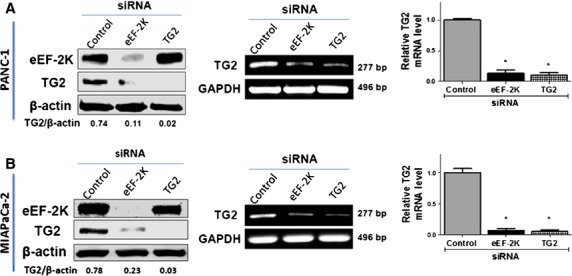
Eukaryotic elongation factor-2 kinase (eEF-2K) regulates TG2 expression in pancreatic cancer (PaCa) cells. (**A** and **B**) Knock-down of eEF-2K by siRNA down-regulates TG2 expression in PANC-1 (**A**) and MIAPaCa-2 cells (**B**). (Left panel) Cells were transfected with indicated siRNAs, and cell lysates were subjected to Western blot analysis. β-actin was used as loading control. (Middle panel) The total RNA was extracted after cells treatment, and the transcript levels of TG2 were determined by standard RT-PCR as described in Materials and Methods. GAPDH was used as loading control. (Right panel) Histograms show the relative TG2 mRNA levels. *P < 0.05 *versus* control cells. All experiments were independently repeated three times.

Tissue transglutaminase (TG2) is implicated in regulation of cell attachment, interactions of the cells with the surrounding ECM, motility and invasion, and is considered as a bad prognostic factor in different cancers, including PaCa 27,33–35. We previously showed that, in addition to eEF-2K, rottlerin down-regulates TG2, which is overexpressed in PaCa cells 36,37. We show here that eEF-2K regulates TG2 at the transcription level as siRNA-mediated silencing of eEF-2K markedly down-regulates the basal expression of TG2 protein and mRNA levels in PANC-1 cells, as demonstrated by Western blot and RT-PCR analysis, respectively (Fig. 4A). A similar trend was observed in MIAPaCa-2 cells (Fig. 4B). Considering the function of TG2 in cell adhesion, and its role in promoting the motility/survival signalling pathways on the cell surface 34,38, it is suggested that the inhibition of the invasion after knock-down of eEF-2K is mediated, in a part, through TG2 down-regulation, and the eEF-2K/TG2 axis promotes PaCa cells invasion.

### eEF-2K regulates Src activity through TG2 in PaCa cells

**Figure 5:**
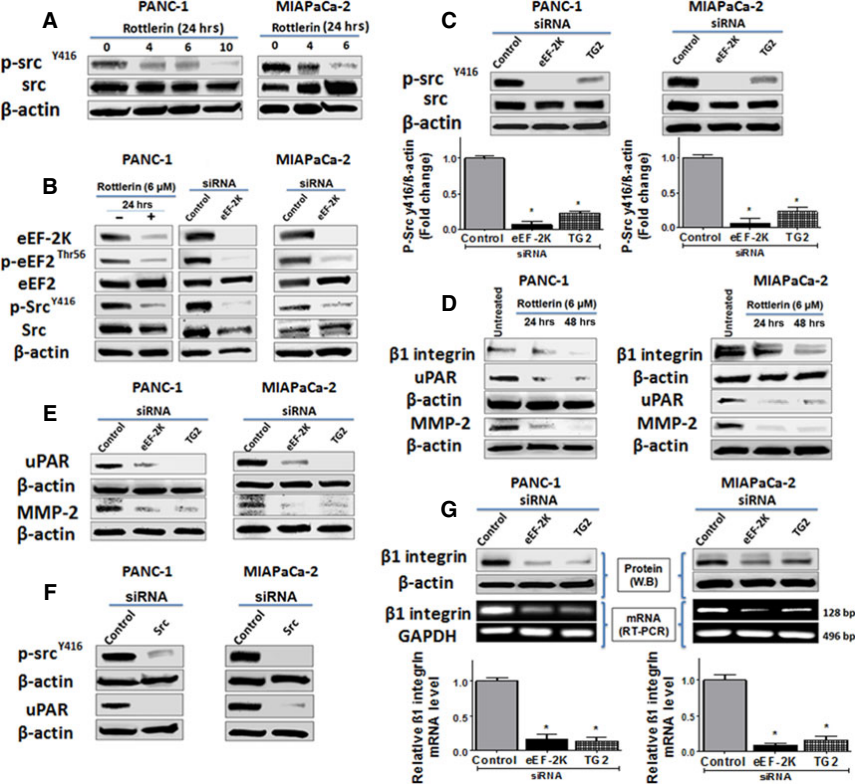
The downstream molecular effects of rottlerin and specific siRNA-mediated knock-down of eukaryotic elongation factor-2 kinase (eEF-2K) and TG2 on invasion biomarkers in pancreatic cancer (PaCa) cells. (**A** and **B**) Rottlerin treatments decrease the expression of p-Src (Tyr-416) concentration-dependently (**A**). Both rottlerin and siRNA-mediated knock-down of eEF-2K are associated with similar molecular downstreams (**B**). (**C** and **E**) Expression of active Src and uPAR/MMP-2 after knock-down of eEF-2K or TG2 by their corresponding siRNAs, in PANC-1 and MIAPaCa-2 cells. The histograms show the relative p-Src (Tyr-416) expression after indicated treatments. (**D**) Rottlerin treatments decrease β1 integrin and uPAR MMP-2 expressions in PaCa cells. (**F**) Src knock-down is associated with a marked down-regulation of uPAR expression. Cells were incubated with or without indicated rottlerin concentrations, for indicated times, or transfected with indicated siRNAs, and cell lysates were subjected to Western blot analysis. β-actin was used as a loading control. (**G**) Expression of β1 integrin after knock-down of eEF-2K or TG2 with their corresponding siRNAs. (Upper panel) Cells were transfected with indicated siRNAs, and cell lysates were subjected to Western blot analysis. β-actin was used as a loading control. (Lower panel) The total RNA was extracted after cells treatment, and the transcript levels of β1 integrin were determined by standard RT-PCR as described in Materials and Methods. GAPDH was used as loading control. The histograms show the relative β1 integrin mRNA levels. Three independent experiments were performed with similar results, and representative data are shown.

Increased Src activity often correlates with the malignant and metastatic potential of many tumours including PaCa 39. More than 60% of PaCa display increased Src activity*,* which is associated with poor prognosis; as it cooperates with oncogenic Ras to accelerate PDAC onset by increasing genomic instability 40. Src activity plays critical roles in adhesion, migration and invasion as well as proliferation of cancer cells; thus, Src is of considerable interest as an attractive molecular target for cancer therapy 9,41,42. Src activation is associated with the phosphorylation at Tyr-416 and dephosphorylation at the negative regulatory site Tyr-527 43. To further elucidate the potential molecular mechanisms by which eEF-2K mediates invasion of PaCa cells, we examined the possible involvement of Src inhibition, by down-regulation of eEF-2K *via* rottlerin and siRNA. We found that rottlerin treatments and eEF-2K siRNA led to significant reductions in Src phosphorylation (at Tyr-416) in PANC-1 and MIAPaCa-2 cells (Fig. 5A and B). Both rottlerin and siRNA treatments induced eEF-2K down-regulation, inhibition of eEF2 phosphorylation (at Thr-56), a well-known downstream substrate of eEF-2K, with subsequent inactivation of Src (down-regulation of phospho-form at Tyr-416; Fig. 5B). The focal adhesion kinase (FAK) is recruited, in association with Src, as a major component of an integrin-dependent signalling pathway 10,44. We also found a marked reduction in the active FAK (p-Tyr-397) after down-regulation of eEF-2K/TG2 and rottlerin treatment (data not shown). Because TG2 expression is regulated by eEF-2K (Fig. 4), we next investigated the role of TG2 in regulating Src activity. As shown in Figure 5C, knock-down of TG2 also significantly reduced the level of active Src in PANC-1 and MIAPaCa-2 cells. Overall, these results suggest that Src is a downstream mediator of eEF-2K, and its activity is regulated through eEF-2K/TG2 axis in PaCa cells.

### eEF-2K down-regulation suppresses β1 integrin and uPAR signalling

Both integrins and uPAR are important mediators of cell invasion 45,46. Beside performing an adhesive function, upon binding to ECM proteins, integrins exhibit transduction of intracellular signals, including activation of tyrosine kinases (*e.g*. Src) and tyrosine phosphorylation of downstream substrates 47,48. In addition, integrin clustering can initiate intracellular signalling events that promote cell proliferation, survival and migration in both normal and tumourigenic cells 49. β1 integrin is known to induce Src activity through the recruitment and activation of signalling FAK/Src kinase complex 46. Importantly, integrin-dependent signalling also regulates the uPAR-mediated activity 50,51. uPAR is a cellular surface receptor that tightly binds to a serine protease uPA, which principally mediates extracellular proteolysis, cell migration, adhesion and mobility 12. uPA and uPAR are known to be overexpressed in mesenchymal and epithelial origin of various cancer and tumour cells and are required for tumour invasion and metastasis 45,52. Plasmin activated by uPA can break down ECM directly or degrade the ECM indirectly through activation of pro-MMPs 53. Although plasmin has been shown to principally activate MMP-1, -3 and -9, increasing evidence proves that uPA/plasmin can activate pro-MMP-2 and thereby promoting tumour invasion and metastasis 53,54. Thus, we examined whether β1 integrin and uPAR/MMP-2 are involved in eEF-2K/TG2 mediated invasion process by inhibiting eEF-2K/TG2 axis by rottlerin and knocking down eEF-2K and TG2 separately. Rottlerin treatments resulted in down-regulation of β1 integrin/uPAR/MMP-2 expression, in both PANC-1 and MIAPaCa-2 cells (Fig. 5D), suggesting that the suppressive effect of rottlerin on cellular invasion could be mediated through its inhibitory effect on β1 integrin/Src/uPAR/MMP-2 signalling. We also found that silencing of eEF-2K and TG2 led to marked decreases in the levels of uPAR and MMP-2 proteins (Fig. 5E), further supporting the link between eEF-2K/TG2 axis and β1 integrin/uPAR expression, and related ECM interactions. Indeed, TG2 can be expressed on the cell membrane in association with integrins and serves as a co-receptor for integrin-mediated cell survival signalling 38, and regulates cell–matrix interactions 34. We also demonstrated that siRNA-mediated knock-down of Src significantly decrease uPAR expression (Fig. 5F). Because Src- and uPAR-mediated activity requires integrin-dependent signalling, we next examined the expression of β1 integrin after silencing eEF-2K and TG2 genes. Our results show that knock-down of eEF-2K and TG2 resulted in marked decreases in the expression of β1 integrin at both protein and mRNA levels in PANC-1 cells, with a similar trend obtained in MIAPaCa-2 cells (Fig. 5G). Overall, these findings suggest that eEF-2K/TG2 signalling plays a role in regulating ECM networks and the invasive phenotype of PaCa cells.

### Rottlerin suppresses TG2/β1 integrin/Src/uPAR/MMP-2 signalling through inhibition of eEF-2K expression

**Figure 6:**
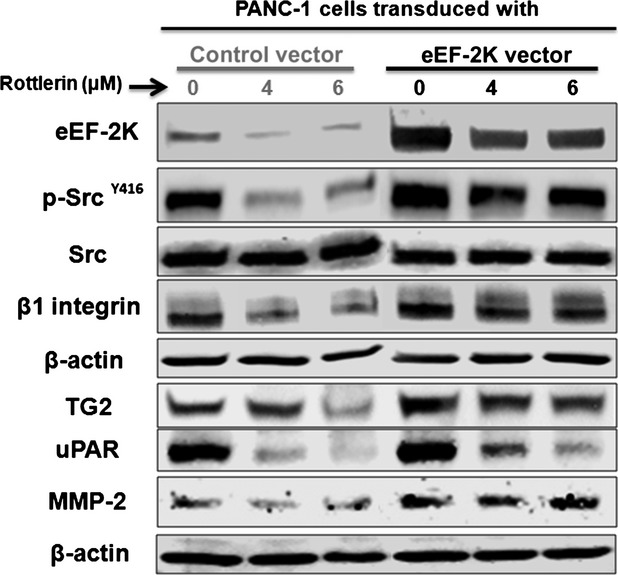
The effect of eukaryotic elongation factor-2 kinase (eEF-2K) overexpression on rottlerin-mediated downstream molecular effects in PANC-1 cells. The cells were stably transfected with pCDH-eEF-2K lentiviral vector or empty control vector, treated with indicated rottlerin concentrations for 24 hrs, and cell lysates were subjected to Western blot analysis. β-actin was used as a loading control. Three independent experiments were performed with similar results, and representative data are shown.

To further support the notion that down-regulation of eEF-2K plays a major role in rottlerin-induced suppression of invasion-mediating machineries, we overexpressed eEF-2K in PANC-1 cells and examined the expression of TG2, β1 integrin, active Src, uPAR and MMP-2 after rottlerin treatment. The results in Figure 6 show the higher basal expressions of TG2, active Src, β1 integrin, uPAR and MMP-2 in correlation with high eEF-2K expression. Consistently, eEF-2K overexpression rescues the cells from rottlerin-induced down-regulation of these invasion-mediating biomarkers, when compared with the marked reductions in their levels after the same rottlerin treatments in control vector-transduced cells. Overall, these results demonstrate a novel signalling of TG2/β1 integrin/Src/uPAR/MMP-2, which is mediated by eEF-2K, and indicate that rottlerin is a potent inhibitor of such signalling.

### eEF-2K/TG2 axis regulates EMT through the zinc finger transcriptional regulators in PaCa cells

**Figure 7:**
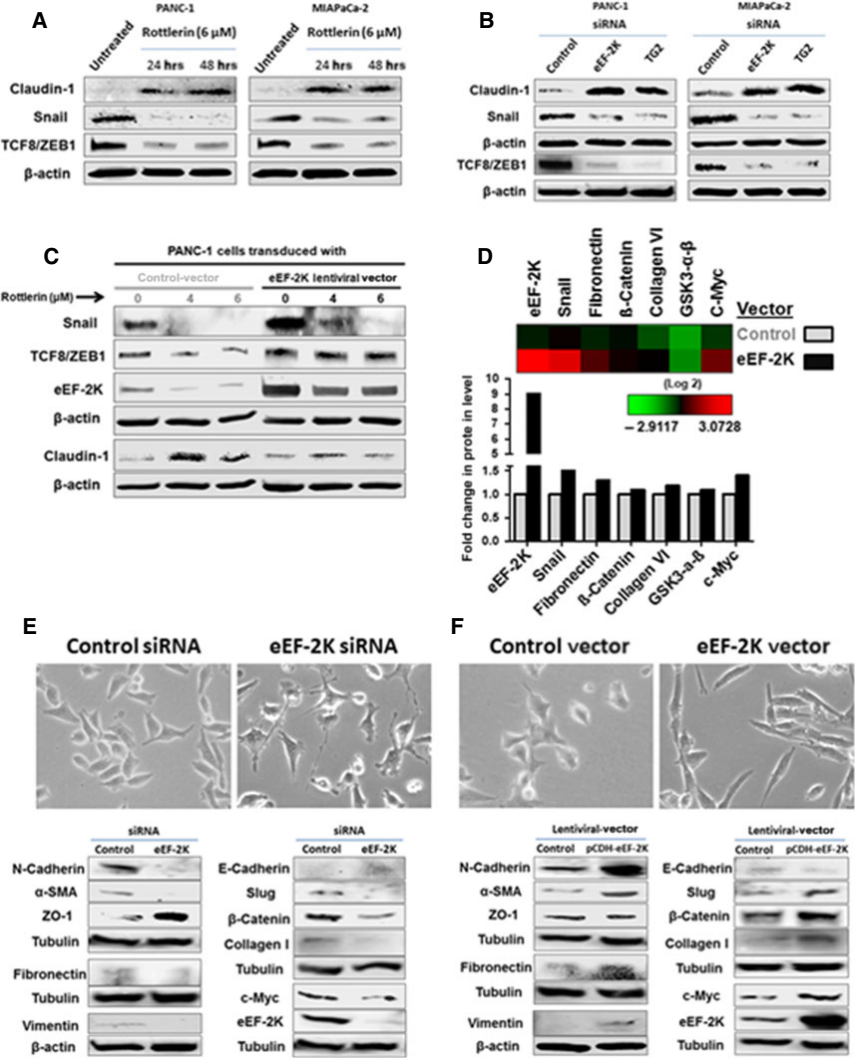
The effect of rottlerin treatment and specific siRNA-mediated knock-down of eukaryotic elongation factor-2 kinase (eEF-2K) and TG2 on epithelial–mesenchymal transition (EMT) process in pancreatic cancer (PaCa) cells. Rottlerin treatment (**A**), and siRNA-mediated knock-down of eEF-2K or TG2 (**B**), significantly increase the expression of EMT tight junction protein, claudin-1, and decrease the zinc finger ZEB1 transcriptional factors, TCF8 and Snail. The cells were incubated with or without indicated concentration of rottlerin, for indicated times, or transfected with indicated siRNAs, and cell lysates were subjected to Western blot analysis. β-actin was used as a loading control. (**C**) The effect of eEF-2K overexpression on rottlerin-mediated effects on EMT process in PANC-1 cells. The cells were stably transfected with pCDH-eEF-2K lentiviral vector or empty control vector, treated with indicated rottlerin concentrations for 24 hrs, and cell lysates were subjected to Western blot analysis. β-actin was used as a loading control. (**D**) Heat map clustered from RPPA analysis revealed an array of some EMT-drivers proteins in PANC-1 cells stably transfected with pCDH-eEF-2K lentiviral vector or empty control vector. The histograms demonstrate the normalized fold changes of indicated proteins levels. (**E** and **F**) eEF-2K induces EMT phenotype in PaCa cells. (Upper pannel) Cell morphological changes associated with eEF-2K knock-down (**E**) or overexpression (**F**) in PANC-1 cells after 48 hrs culture in medium, are shown with phase-contrast microscopy (original magnification ×100). (Lowe pannel) Western blot analysis of epithelial marker (E-cadherin, ZO-1), mesenchymal markers (N-cadherin, Slug, Vimentin, fibronectin, α-SMA, β-Catenin and Collagen type I) and relative expression of eEF-2K and c-Myc after siRNA-mediated knock-down of eEF-2K (Lower left panel), or eEF-2K plasmid-induced overexpression of eEF-2K (Lower right panel). Tubulin or β-actin served as a loading control. Three independent experiments were performed with similar results, and representative data are shown.

A prerequisite of the ability of a cancer cell to undergo metastasis into distant tissues is to penetrate the surrounding ECM 10. EMT is a critical process that requires a loss of cell–cell adhesion, as well as the acquisition of a mesenchymal motile and invasive phenotype 55. It is a transcription factors-regulated process that is implicated in the progression of primary tumours towards metastasis 56. Thus, we first investigated if rottlerin modulates EMT to interfere with the invasiveness of PaCa cells. As shown in Figure 7A, rottlerin treatment led to increase in the expression of claudin-1, and decrease in the level of the zinc finger ZEB1 transcriptional regulators, Snail and transforming growth factor-8 (TCF8), that act as repressors of the cell–cell junctions key mediator, E-cadherin 33. TCF8 is one of the ZEB1 family transcription factors that induce EMT, and triggers epithelial dedifferentiation by impairing the expression of E-cadherin 16,17. Snail is a transcriptional repressor of both E-cadherin 33 and claudins 57. The EMT tight junction protein, claudin-1, is known to be expressed in various types of epithelial cells and plays an important role in epithelial cell polarity. The expression of claudin-1 was markedly decreased in various types of PaCa cell lines compared with normal pancreatic duct epithelial cells 58. Studies have shown that the increased TG2 expression in advanced invasive cancer cells has been implicated in the modulation of cell adhesion, acquisition of EMT and cancer metastasis 27. To explore whether eEF-2K/TG2 signalling plays a role in modulation of EMT process for maintaining the invasive phenotype of PaCa cells, we knocked down eEF-2K and TG2 genes using siRNA. Our results demonstrated that down-regulation of either eEF-2K and TG2 genes significantly reduced the expression of TCF8/ZEB1 and Snail, with concomitant induction of claudin-1 expression, indicating the effective inhibition of EMT 15 in both PANC-1 and MIAPaCa-2 cells (Fig. 7B). These results suggest that eEF-2K/TG2 axis induces the acquisition of the EMT of PaCa cells.

### eEF-2K expression rescues PaCa cells from rottlerin-induced impairment of EMT-promoting signalling

To further validate the role of eEF-2K in EMT regulation and in rottlerin-induced inhibition of PaCa invasion, we overexpressed eEF-2K gene in PANC-1 cells. Expression of eEF-2K transgene in PANC-1 cells genomic DNA displays enhanced expression of TCF8/ZEB1 and Snail, when compared with empty vector-transduced cells (Fig. 7C). Consistently, high eEF-2K expression suppresses rottlerin-mediated inhibition of TCF8/ZEB1 and Snail, as well as decreases rottlerin-mediated induction of claudin-1. In conclusion, rottlerin treatment-correlated inhibition of EMT markers and induction of epithelial markers is most likely mediated *via* inhibition of eEF-2K-dependent signalling in PaCa cells.

### eEF-2K expression promotes acquisition of EMT phenotype and characteristics

Through our analysis by RPPA, we noticed the correlation between eEF-2K expression and the expression levels of some proteins that are involved in EMT attainment (Fig. 7D). Cancer cells that undergone EMT loose cell–cell contacts, acquire mesenchymal properties to develop migratory and invasive abilities. Thus, we next investigate the morphological alterations after eEF-2K overexpression, to ask if up-regulation of eEF-2K is sufficient to promote the characteristic mesenchymal morphologic changes. It is worth mentioning that PANC-1 cells cell line originated basically from a primary tumour in the pancreatic duct of an epithelioid carcinoma patient. Thus, PANC-1 cells show relative epithelial cell-like morphology and is poorly differentiated 59,60 (Fig. 7E). In our study, eEF-2K-transduced PANC-1 cells demonstrated acquisition of elongated spindle-shaped fibroblast-like morphology that enables the cells to invade ECM (Fig. 7F). The morphological changes during EMT are driven by a number of molecular alterations, including loss or decrease in epithelial cell markers (*e.g*. E-cadherin and claudins/occludins) and *de novo* expression of mesenchymal markers (*e.g*. N-cadherin, Vimentin, Slug and Fibronectin) 61. We further investigated the expression of EMT makers after eEF-2K silencing and overexpression. Indeed, PANC-1 cells exhibit low levels of E-cadherin that could be owed to the high basal expression of ZEB1/Snail which impair its expression. Loss of E-cadherin is considered to be a fundamental event in EMT in tumour progression 18,19. In addition to the induction of the transcription factors Snail and TCF8/ZEB1, eEF-2K-vector cells expressed high levels of mesenchymal markers, N-cadherin, Vimentin, Slug, Fibronectin, β-Catenin, α-smooth muscle actin (α-SMA) and Collagen 1 as well as the transcription factor c-Myc. Conversely, epithelial cell markers, as ZO-1 and E-cadherin, were reduced after eEF-2K overexpression (Fig. 7D and F). In contrast, knock-down of eEF-2K induced the reverse effects (Fig. 7E), maintaining the features of the epithelial cells that exhibit apical–basal polarity and close linkage to adjacent cells by cell adhesion molecules and junctions. ZO are epithelial tight junction proteins that represent the principle components of the junctional complex linking neighbouring epithelial cells and maintaining their polarity. The tight junctions function as suppressors of proliferation and transformation, and their expression is often deregulated in cancer tissues 62. α-SMA is a highly conserved protein that is involved in cell motility and EMT 63. Importantly, c-Myc expression in human mammary epithelial cells was associated with a dramatic change in cell morphology, and inducing EMT characteristics along with reverse correlation with E-cadherin expression 64. The up-regulations of the glycoprotein, Fibronectin and the intermediate filament, Vimentin, are markers of EMT and have been correlated with metastasis 64. Slug (Snail-2) is E-cadherin transcriptional repressor that, together with Snail-1 promotes formation of β-Catenin/TCF4/LEF-1 transcription complexes that initiate EMT in different types of cancers 65. Recently, Snail expression in PaCa cells was found to promote collagen I production by pancreatic stellate cells (PSCs), promoting fibrosis. Moreover, it was found that β-catenin/TCF4 binds directly to the ZEB1 promoter and activates its transcription in colorectal carcinoma triggering an EMT profile 66. Overall, our results indicate, for the first time, that eEF-2K is tightly bound to EMT program, that might explain the enhanced migratory and invasiveness capacity of the PaCa cells overexpressing eEF-2K.

### eEF-2K induces Collagen expression in PaCa cells

The ECM components provide the pivotal microenvironment for tumour development/progression, including PaCa, which is notoriously characterized by a pronounced fibrotic reaction consisting of proliferating stromal cells together with collagen-rich ECM 67. Collagens are the major structural ECM proteins, and its interaction with the cells controls cellular proliferation, migration and invasion through integrin and discoidin domain receptors. In fact, enhanced expression/deposition of collagens is associated with the development/progression of different tumours 68. PaCa is characterized by excessive deposition of type I collagen, promoting cells motility and up-regulating mesenchymal markers. In addition, Collagen VI is another prominent promoter of tumour growth that is frequently overexpressed in human cancers. Increasing evidence indicate that collagen VI directly acts on the Akt/GSK-3-β/β-catenin/TCF/LEF axis, enhancing the production of protumourigenic factors and inducing EMT 69. Thus, we further determined the alterations in Collagens in response to the change of eEF-2K expression, and we found an elevated expression of collagen I and VI levels after overexpression of eEF-2K (Fig. 7D and F), further supporting the regulatory role of eEF-2K on collagens-regulated EMT, migration and invasion of PaCa cells.

### eEF-2K-mediated c-Myc expression might be involved in eEF-2K-induced EMT characteristics

In primary and metastatic pancreatic adenocarcinomas, c-Myc has been found to be overexpressed 70. Recently, inhibition of endogenous c-Myc was found to reduce the motility, invasion, and proliferation of some carcinomas 71. More importantly, c-Myc-induced mammary tumours demonstrated altered gene expression profiles reflecting the induction of features of EMT, suggesting the link between c-Myc expression and EMT genes and characteristics 72. In addition, the transduction of immortalized human mammary epithelial cells with exogenous c-Myc induced a dramatic change in cell morphology from regular epithelial cuboidal morphology into fibroblastoid morphology, with loss of E-cadherin transcriptional expression as well as increased the solubility of Vimentin and Fibronectin 64. We showed before that eEF-2K regulates the expression of several pro-tumorigenic proteins in breast cancer cells, including the transcription factor, c-Myc 26. In this study, we showed that c-Myc levels elevated after eEF-2K overexpression (Fig. 7D and F), and decreased after eEF-2K knock-down (Fig. 7E). We also found a correlation between TG2 levels and c-Myc expression (data not shown). Through our other studies, we found that c-Myc, has two predicted binding sites on TGM2 (the gene symbol of its protein product, TG2; unpublished data). Thus, it is suggested that eEF-2K regulates TG2 through activation of c-Myc. Because of the close association between eEF-2K/TG2/c-Myc levels and EMT profiling, we assumed that the transcription activity of c-Myc could account for the activation of the genes responsible for highly invasive phenotype including mesenchymal markers. However, further investigation is required to further prove the exact role of c-Myc in EMT of PaCa cells.

**Figure 8:**
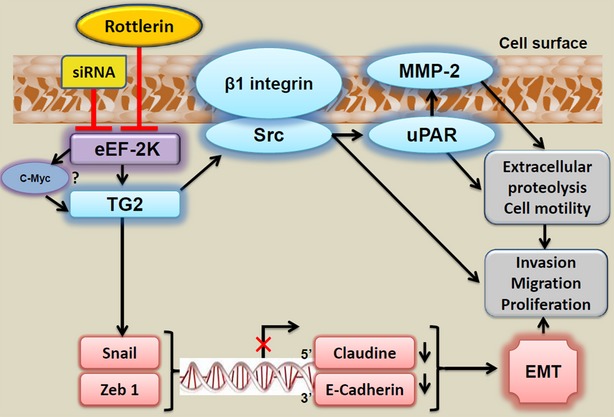
The postulated molecular regulation of eukaryotic elongation factor-2 kinase (eEF-2K)/TG2 pathway to the invasion-promoting signalling in pancreatic cancer (PaCa) cells. Rottlerin- or siRNA-induced down-regulation of eEF-2K constitutively suppresses the signalling involving TG2/β1 integrin/Src/uPAR/MMP-2-mediated degradation of ECM and enhancement of cell invasion/migration. eEF-2K regulates TG2, probably through c-Myc. The expression of c-Myc in many tumours (*e.g*. mammary tumours) induces altered gene expression reflecting an epithelial–mesenchymal transition (EMT) profile. eEF-2K/TG2–mediated signalling cascade plays an important role in the survival mechanisms controlling cellular proliferation, motility and invasion and stimulates the expression of the zinc finger transcriptional factors (Snail and TCF8/ZEB1). These mesenchyme markers repress gene transcription of epithelial markers (claudins and E-cadherin) facilitating EMT, further supporting the motility and invasiveness of PaCa cells.

Figure 8 depicts a summary of suggested rottlerin- and eEF-2K down-regulation-mediated mechanisms of suppression of the PaCa cells invasion.

## Discussion

The major hallmark of PDAC is extensive local tumour invasion and early systemic dissemination, contributing to <4% 5-year survival rate 31. Metastasis is the major cause of patient mortality in almost all cancers 3,7,8. The poor prognosis of PDAC is attributed to its resistance to conventional therapies, as well as its highly invasive and aggressive nature, since 80% of PaCa patients have already metastatic lesions at the time of diagnosis 3,73. PDAC’s aggressive biology, desmoplastic reaction, high metastatic potential and refractory to traditional therapy warrant the development of novel and effective targeted therapies for PaCa, to prevent invasion and metastasis, and to prolong the poor patient survival.

Our current study provides the first evidence regarding the potential role of eEF-2K in mediating PaCa invasion and metastasis. It also provides insight into the mechanisms exhibited by eEF-2K to promote the invasive phenotype of PaCa cells, through a novel signalling axis involving TG2/β1 integrin/Src/uPAR/MMP-2 and induction of EMT. Although rottlerin has been used as a specific PKC-δ inhibitor 74,75, the recent studies, including ours, suggest that rottlerin can modulate cancer progression through its effects on several biological cellular targets other than PKC-δ 20,76,77. Taking into account such role of eEF-2K in PaCa biology, our results also indicate that targeting of eEF-2K could represent a potential novel therapeutic approach in controlling metastasis and progression in PaCa.

The ability of the cancer cells to invade the basement membrane into surrounding tissues, blood and lymphatic vessels is one of the essential steps and is a prerequisite for local tumour progression and metastatic spread 31. As cancer cells invade/metastasize into new tissues, they penetrate and attach to the target tissue’s basal matrix 47. In fact, little is known, if any, regarding the role of eEF-2K in PaCa biology. However, the present study shows that eEF-2K-mediated signalling may promote the ability of PaCa cells to undergo invasion, as silencing eEF-2K expression severely impaired the invasion/migration ability of PaCa cells. This study also explored the downstream signalling that might be attributable as major mediators of eEF-2K-dependent effects. We showed that eEF-2K regulates TG2 expression to regulate the invasion of PaCa cells. Because c-Myc is regulated by eEF-2K (Fig. 7), and has two predicted binding sites for TGM2 gene promoter (our unpublished data), it is assumed that c-Myc mediates eEF-2K-induced TG2 expression. TG2 has been previously implicated in poor prognosis and low patient survival, and is involved in a variety of cellular processes, such as cell differentiation, death, inflammation, migration and wound healing 27. The metastatic potential of tumour cells has been attributed to expression of high basal levels of TG2 78. Although it is predominantly a cytosolic protein, TG2 can be secreted outside the cell where it regulates cell–matrix interactions 34. It can also translocate to the nucleus, where it associates with pRb, p53 and histones to regulate certain cellular functions 79. TG2 also can be expressed on the cell membrane in association with members of the integrin family and serves as a co-receptor for integrin-mediated cell survival signalling 38. TG2 promotes the interaction between cell surface integrins and fibronectin to support cell adhesion, motility and invasion 34. The cell invasion and attachment to the target tissue’s basal matrix is mediated by cell surface receptors (integrins) 47. Integrins are trans-membranous α/β heterodimeric receptors that mediate cell–cell interactions and cell attachment to ECM 48, and they serve as receptors for some ECM proteins (*e.g*. Fibronectin, Vitronectin, Laminin and Collagen) 80. Collagens present in the extracellular spaces and basement membranes are recognized by specific integrins 81. Importantly, integrins (β1, β4 and β5) can exist in complex with TG2 in cancer cell membranes 80. Our current study shows that eEF-2K/TG2 knock-down led to a remarkable fall in β1 integrin expression (Fig. 5G), with a marked inhibition of the non-receptor protein tyrosine kinase Src phosphorylation (activation; Fig. 5C). Src family is one of the most important mediators of tumour progression and is involved in cancer cell proliferation, angiogenesis, invasion and metastasis. Importantly, elevated Src activity in PaCa contributes to K-ras-dependent tumorigenesis, and Src inhibition suppresses growth of Ras/Src-driven pancreatic tumours 40. Src also has been implicated in cell/cell contacts, migration, invasiveness and controlling signal transduction downstream of a variety of cell surface receptors including the integrins 44,82. In addition to the role of integrins as cell adhesion molecules, their ligation with ECM ligands can generate intracellular signals through the recruitment and activation of non-receptor tyrosine kinases, Src and FAK that form a dual kinase complex 46. The activation and co-clustering of integrin-associated non-receptor protein kinases, by ECM–integrin interactions, initiate signals that transduced into cells to regulate biological cell migration, motility, survival, differentiation and proliferation 46–48.

Urokinase-type plasminogen activator receptor has been shown to be up-regulated in cancer cells and to play a critical role to regulate the cells-ECM interactions, promoting its degradation and turnover through the plasminogen activation cascade 31,45. Indeed, uPAR and integrins have been shown to co-operate in migration of many cancer cells including monocytes, fibrosarcoma HT1080, melanoma, MCF-7 breast cancer and fibroblasts 45. At the leading edge of migrating cells, the uPAR binds and activates uPA; active uPA proteolytically converts inactive plasminogen to active plasmin (fibrinolysin), a trypsin-like enzyme that induces pericellular proteolysis in fibrin and fibrinogen 13. Plasmin contributes to the systematic activation of the coagulation cascade and can break down ECM directly or degrade the ECM indirectly through activation of pro-MMPs. MMP-2, rather than MMP-9, was activated in the metastatic PaCa, and it is secreted as an inactive zymogen and requires distinct activation processes 53. MMP-2 can be activated through binding of pro-uPA to its receptor, uPAR 83, activation of plasminogen/plasmin on the cell surface, and localization these enzymes to focal contact sites 53,84. The activity of uPA/uPAR-system correlates with the increased cellular proliferation, migration and invasion, affecting the malignant phenotype of cancer 12,13. Although the involvement of uPA cascade in MMP-2 activation was previously reported in metastatic PaCa BxPc3 cells 53, we showed for the first time that the expression pattern of these proteins is regulated by eEF-2K/TG2/β1 integrin/Src axis, in the metastatic PANC-1 and MIAPaCa-2 cells (Fig. 5). We presented an interesting hypothesis whereby eEF-2K and TG2 form a mutual positive regulatory loop in modulation of β1 integrin-dependent signalling to promote the invasive phenotype of PANC-1 and MIAPaCa-2 cells, facilitating the tumour progression in these cells. Considering its potent effect in inhibition of eEF-2K and correlated signalling, rottlerin could be used to suppress the invasive phenotype of PaCa cells.

Epithelial–mesenchymal transition is one of the important cancer drivers that enhance metastasis of primary tumours into the surrounding tissues 56. The progression of the EMT program is regulated by a series of intracellular signalling molecules; including transcription factors such as the zinc finger proteins of the δEF1/ZEB, Snail/Slug family, Twist and SIP1, that have been implicated in the transcriptional repression of E-cadherin. These repressors also trigger the EMT program by repressing genes encoding claudins, cytokines, integrins, mucins and occludin proteins, thereby promoting EMT 27, and pre-disposing tumour cells to invasion and metastasis 56. Our results show that the cells which express high level of eEF-2K exhibited acquisition of elongated fibroblast-like morphology, along with gain in the expression of mesenchymal markers, N-cadherin, Vimentin, Slug, Fibronectin, Collagen 1 and α-SMA, and reduction/loss of epithelial cell markers, E-cadherin and ZO-1. Knock-down of eEF-2K induced the reverse effects (Fig. 7). In addition, we show that eEF-2K/TG2 axis regulates the transcriptional mediators of EMT and that targeting of eEF-2K/TG2 resulted in marked suppressions of the prime factors, TCF8/ZEB1 and Snail, with obvious up-regulation of claudin-1 expression, in both PANC-1 and MIAPaCa-2 cells (Fig. 7B). Furthermore, our current results revealed the increase in c-Myc as well as TG2 expression in eEF-2K overexpressed cells, with reverse effects occurred after silencing eEF-2K. Indeed, c-Myc expression was previously found to induce a fibroblastoid-like morphological changes in human mammary cells, along with loss of E-cadherin and increase in Vimentin and Fibronectin solubility 64. Furthermore, TG2 was recently shown to be recruited (in association with NF-kB) to the promoter sequence of Snail and leading to its transcriptional regulation 33. Importantly, a marked low expression of claudin-1 was previously observed in poorly differentiated human PaCa (*e.g*. PANC-1 and BxPC3), as well as moderately and well-differentiated PaCa (*e.g*. HPAF-II and HPAC) 58. Down-regulation of other tight junction proteins was also observed in PaCa cells during EMT 85.

It is worth mentioning that the PDAC fibrotic reaction, which can account for more than 80% of the tumour mass, has been shown to limit the delivery of therapeutics, and contribute to cell survival and drug resistance 67. EMT-induced PaCa cells might promote invasive ability not only by increasing cell motility but also by enhancing collagen internalization, whose initial step is binding of collagen to α2-β1-integrin membrane receptors that play a role in the internalization/uptake process in fibroblasts 11. Recently, Snail was found to promote *in vivo* pancreatic fibrosis through activation of PSCs which, in turn, express α-SMA and depositing excess type I collagen 67. Pancreatic carcinomas exhibit severe invasive malignant phenotype and fibrotic response, with high levels of collagen I deposition, *via* increasing N-cadherin expression 86. Interestingly, E-cadherin gene expression was markedly repressed in PANC-1 and BxPC3 cells after cultured on type I or type III collagen, or after overexpression of activated c-Src 87.

In conclusion, because of the involvement of eEF-2K in EMT program along with integrin β1/ECM/Collagens signalling, and considering the eEF-2K-induced Src activation, eEF-2K represents a novel potential therapeutic target. Since overexpression of eEF-2K in PaCa is suppressed by developmental siRNA based-therapies, identification of specific eEF-2K inhibitors could provide a significant tool for blocking PDAC local invasion. To better understand the role of eEF-2K signalling in PaCa progression, its expression in patients’ tumours samples should be analysed, its association with downstream pathways must be investigated, and its clinical significance in survival and drug resistance should be determined.
